# Resilience and mental health among perinatal women: a systematic review

**DOI:** 10.3389/fpsyt.2024.1373083

**Published:** 2024-07-22

**Authors:** Mohammedamin Hajure, Solomon Seyife Alemu, Zakir Abdu, Gebremeskel Mulatu Tesfaye, Yadeta Alemayehu Workneh, Aman Dule, Mustefa Adem Hussen, Lema Fikadu Wedajo, Wubishet Gezimu

**Affiliations:** ^1^ Department of Psychiatry, Maddawalabu University, Shashemene, Ethiopia; ^2^ Department of Midwifery, Maddawalabu University, Shashemene, Ethiopia; ^3^ Department of Psychiatry, College of Health Sciences, Mattu University, Mattu, Ethiopia; ^4^ Department of Nursing, College of Health Sciences, Mattu University, Mattu, Ethiopia; ^5^ Department of Midwifery, College of Health Sciences, Mattu University, Mattu, Ethiopia; ^6^ Department of Midwifery, Institute of Health Sciences, Wollega University, Nekemte, Ethiopia

**Keywords:** mental health, associated factor, perinatal, women, resilience

## Abstract

**Objective:**

This review aimed to assess the current evidence on the relationship between resilience and mental health employed in response to the impacts of mental health.

**Method:**

This review was conducted in accordance with the Preferred Reporting Items for Systematic Review and Meta-analysis (PRISMA). The protocol of this review was registered on the International Prospective Register of Systematic Reviews (PROSPERO: CRD42023470966). Three authors searched peer-reviewed articles using several electronic databases, including Scopus, PubMed/MEDLINE, Psych Info, EMBASE, and Web of Science, from September to October 2023 and included all the studies from any time until November 1, 2023. The review included all eligible quantitative observational and qualitative studies, irrespective of geographical boundaries.

**Result:**

Depression, anxiety, and post-traumatic stress disorders were found to be the most common, but not the only, mental health disorders during the perinatal period, and higher maternal resilience during perinatal periods was found to reduce mental health disorders. It was also found that pregnant women were more resilient to mental health disorders than postpartum women. Tolerance of uncertainty and a positive cognitive appraisal, women’s self-behavior and family functioning, and protective psychosocial resources such as dispositional optimism, parental sense of mastery, self-esteem, gratitude, and forgiveness were found to be the most common mechanisms of resilience among perinatal women. Older age, having an adolescent partner, family income, and distress were found to affect resilience.

**Conclusion:**

Noting that women’s resilience is an important tool to prevent perinatal mental health disorders, maternal healthcare providers need to counsel perinatal women on resilience-boosting mechanisms, such as applying self-behavior and having social support or close family relationships. It is recommended to counsel or provide psychosocial interventions for the woman’s companion or partner to give strong support for the woman in each of the perinatal periods.

**Systematic review registration:**

https://www.crd.york.ac.uk/prospero/display_record.php?RecordID=470966, identifier CRD42023470966.

## Introduction

Mental health is the backbone of other health, defined as a state of mental well-being that permits individuals to cope with stress conditions, realize their abilities, and be productive in their communities. It’s a fundamental human right of an individual and a milestone in personal, community, and socioeconomic development (1).

Resilience is the ability to bounce back from struggling or challenging life experiences with a successful outcome, mainly through mental, psychological, and behavioral flexibility and adjustment to external and internal demands (2). Every person will experience difficulties or challenges in their life. However, the main concern is the ability to respond to these difficulties without serious harm because it allows them to overcome negative experiences and helps them to learn from them (2, 3).

Nowadays, mental health disorders (MHDs) are a worldwide burden of diseases. As described from the updated data in the 2019 global burden of diseases reports, mental disorders account for more than 14% of years lost due to disability for about 30 years (4). Furthermore, globally, more than 300 million and 280 million individuals suffer from anxiety and depression, respectively (4, 5).

Perinatal mental disorders (PMDs) are mental and behavioral disorders that occur during pregnancy and postpartum period. It affects more than 20% of perinatal women (6). Depression and anxiety are among the common mental disorders that occur during the perinatal period, followed by posttraumatic stress disorder and postpartum psychosis (7). Those mental disorders during the perinatal period may occur as a result of experiences associated with childbirth, neonatal loss, malformation birth, intimate partner violence, previous history of abuse, unwanted pregnancy, complications in labor, and higher perceived stress (8–10).

Mental disorders during the perinatal period result in many devastating impacts on both mothers and their newborns unless they are detected early and managed accordingly (11). The negative consequences of mental disorders include maternal morbidity and mortality associated with increased risk of maternal suicide and impaired motherhood capability that extremely affect the physical, emotional, social, and cognitive development of their newborns (12, 13). Furthermore, perinatal mental disorders have a huge economic burden; for instance, in the United Kingdom (UK), an estimated £75,728 and £34,840 expenditure per woman for perinatal depression and anxiety, respectively, with an aggregate cost of £6.6 billion (14).

Despite the perinatal period being the most challenging time with a lot of psychological, physical, and social changes for women, most women adapt to the situation and care for their family members by accepting the change as exciting and joyous (15). Besides this, several women experience a wide range of negative emotions, which can result in biopsychosocial distress (15, 16). Thus, perinatal resilience is crucial for the woman’s well-being, family balance, adaptation, or acceptance when faced with stressors, challenges, or adversity during the perinatal period (17).

Some of the studies show the impact of resilience in overcoming mental disorders like depression, anxiety, and post-traumatic stress disorders (15, 18–20). As identified in different literature, mechanisms of resilience that help to recover from perianal mental disorders are mastery, optimism, spirituality, social support, adequate financial resources, and a healthy perinatal environment (20–22). In addition to this, cognitive behavioral therapy and mindfulness interventions are also identified as mechanisms for coping with mental disorders during the perinatal period (23). In other ways, various studies assessed resilience specifically for anxiety, depression, and posttraumatic stress disorders in different perinatal periods. Thus, there is no consistent finding on the mechanism of resilience to the perinatal period of mental disorders. Therefore, this review aimed to assess the current evidence on the relationship between resilience and mental health employed in response to the impacts of mental health.

## Materials and methods

### Study protocol and registration

The protocol of this review was registered on the International Prospective Register of Systematic Reviews (PROSPERO) with the accession number (CRD42023470966). This review was conducted in accordance with the Preferred Reporting Items for Systematic Review and Meta-analysis (PRISMA).

### Article search strategies

Three authors searched peer-reviewed articles using several electronic databases including Scopus, PubMed/MEDLINE, Psych Info, EMBASE, and Web of Science, from September to October 2023, and included all the studies from any time until November 1, 2023. In addition, they searched for different relevant gray literature using Google Scholar. Boolean operators ‘OR’ and ‘AND’ were used in between the articles’ keywords and the medical subject heading terms (MeSH). The keywords used were ‘resilience’ OR ‘coping’ AND ‘antenatal’ OR ‘prenatal’ OR ‘during pregnancy’ OR ‘pregnant women’ OR ‘intrapartum’ OR ‘during birth’ OR ‘during labor’ OR ‘during childbirth’ OR ‘postpartum’ OR ‘postnatal’ OR ‘after birth’ AND ‘depression’ OR ‘anxiety’ OR ‘suicide’ OR ‘substance’ OR ‘post-traumatic stress disorder (PTSD)’ OR ‘general psychological distress’.

### Definition of terms

#### Resilience

Since there is no agreed-upon definition of resilience, authors have instead defined it as it is also described as a circular process towards a greater wellbeing in the form of personal growth, family balance, adaptation or acceptance when faced with stressors, challenges, or adversity during the perinatal period (15).

#### Perinatal period

refers to the period between pregnancy and the first 12 months following delivery (24).

### Eligibility criteria

All full-length articles published in English, irrespective of time and geographical boundary, were selected according to Mattos TC. et al.’s PICO/PECO format (Patient/Problem/Population; Intervention/Exposure; Comparison and Outcomes) ideal questions intended to solve a clinical problem (25). Accordingly, this review included all original studies conducted among perinatal (prenatal, intranatal, and postnatal) women (P). The resilience of perinatal women was the exposure (E) status of the review. The outcome (O) interests of this review were the mental health outcomes, including depression, anxiety, suicide, substance use, post-traumatic stress disorder, and general psychological distress. Even if the current review primarily targeted the above-stated mental health conditions, it was not restricted to this but rather included mental health conditions affecting women in the perinatal period, such as sleep disorders, child psychiatric disorders, and lifetime trauma.

In addition, in this review, all published and unpublished studies in the English language that have a clear outcome of interest and are available in full text were included, including all published and unpublished randomized controlled trials (RCTs), observational studies, and qualitative studies. The review included all eligible quantitative observational and qualitative studies, irrespective of geographical boundaries. For the inclusion of qualitative studies, a discussion guide with queries related to mental health should be available. However, it excluded editorials, corrigendum, case studies, reviews, commentaries, conference abstracts, reports, and any articles published in languages other than English that were not published in full-length and utilized designs other than RCT, observational, and qualitative studies.

### Screening and article selection process

Two authors (MAH and WG) conducted the data extraction and article section from October 13 to 16, 2023. The third author (SS) was enlisted to settle disagreements among authors about whether to include or exclude manuscripts from the review process. The full texts of every study that was included were compared again to the specifications, and any discrepancies were resolved by the authors. The data were extracted based on the article’s author name, year of publication, study setting and period, study design, sample size, perinatal period, data collection methods, population demography, magnitude of mental health conditions, mechanism of resilience employed, association of resilience with mental health, and risk impacts or determinants of resilience/future implications. The extracted articles were transferred to Endnote version X9 to manage references and duplications. Then the extracted articles were independently screened by the three authors.

### Critical appraisal of the studies

Two independent reviewers (SS, YA) evaluated the quality of the included studies using the Joanna Briggs Institute (JBI) and the Mixed-Methods Appraisal Tool (MMAT). The reviewers discussed any discrepancies in the evaluation until they could come to a consensus and when a disagreement arose, the third reviewer (ZA) took the study into consideration and mediated a settlement.

The methodological qualities of the quantitative observational studies were appraised using the JBI Systematic Reviews Checklist for Systematic Reviews and Research Syntheses. The modified JBI checklist has nine (9) items to which a response of “yes” or “no” is possible. For this review, the checklist was converted to a scale. A total quality score, ranging from 0 to 18, was obtained by adding the scores of each item, which were assigned a value of 2 (excellent), 1 (good), or 0 (not available, unclear, or poor) (26). In other ways, the Mixed Approaches Appraisal Instrument (27), a critical appraisal instrument, was employed to assess the quality of the mixed studies included because papers using a variety of approaches were included in this study, and they have been validated and demonstrated to have good reliability (28). The MMAT has five quality criteria and wasutilized to evaluate the possibility of bias in the results and to give an insightful description of overall quality. Quality assessment of mixed-methods research was conducted both in the contexts in which the quantitative and qualitative components were employed, and in their own context (29).

## Results

**Figure 1:**
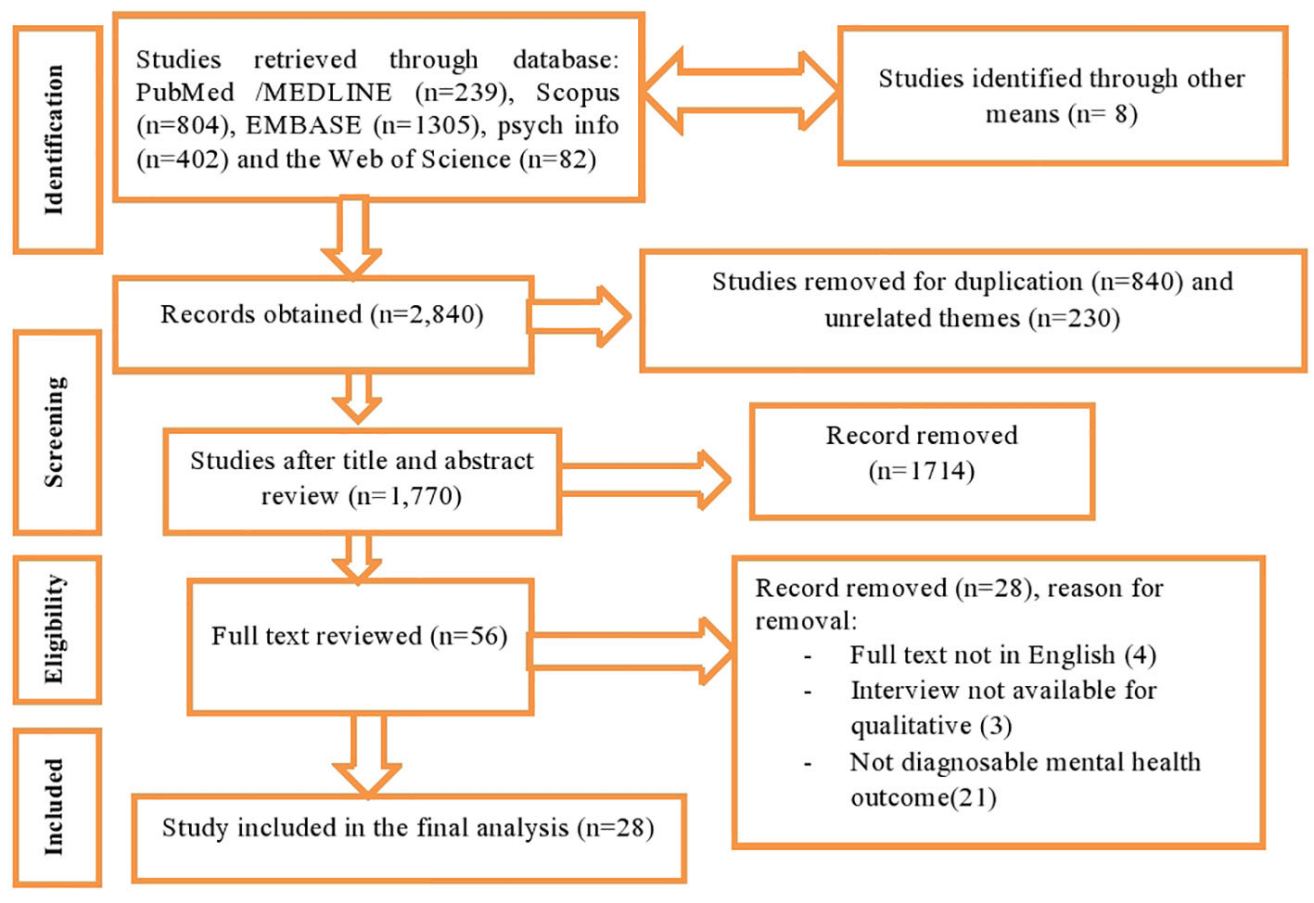
PRISMA flow chart displaying the selection process of identified studies.

In order to find pertinent literature for our review, we employed a variety of databases as search engines, including PubMed/MEDLINE, Scopus, Embase, PsychInfo, and the Web of Science database. Gray literature has also been checked for in other places, such as Google Scholar. As a consequence, 2,840 articles were initially sourced from various sources, including five databases. 1,770 studies were still available for further review after being screened for duplicates and irrelevant themes. Then, 1,714 studies were discarded after title and abstract verification. Afterward, 56 studies had become candidates for full-text review. The authors finally included 28 papers that fulfill the methodological or eligibility requirements to conduct systematic review (**Figure 1**).

### Characteristics of included studies

Regarding the study settings, the majority of the included studies were conducted in developed nations, with Asia following in second. Accordingly, the USA reported nine of the studies, followed by China with seven studies. Additionally, three and two studies came from Australia and India, respectively. For this review, single studies were donated from a variety of other nations, including Spain, Poland, Brazil, Ecuador, Saudi Arabia, Nigeria, and Ethiopia **Table 1**.

**Table 1:** Characteristics of included studies, 2024.

Quantitative studies							
S.N	Author, year of publication	Study setting and period,design, sample,and Perinatal period and data collection methods	Tool to assess resilience and mental health condition	Magnitude of mental health conditions	Resilience mechanism	Association of resilience with mental health	Risk impacts or determinants/future implication
1.	Lubian Lopez et al., 2021 (30)	Spain, during COVID-19, multi-centre cross-sectional survey, 514, During pregnancy, online self-administered questionnaire	EPDS, State-Trait Anxiety Inventory and CDRIS-10	Depression, (35.4%) & state anxiety (44.2%)	Financial support Psychological support	Resilience have moderate negative correlation with depression and anxiety.	Not reported
2.	Werchan et al., 2022 (31)	USA, COVID-19 era, sample of pregnant women (N = 2876) and postpartum women (N = 1536), Pregnant and postpartum period (first 12 months of infant life). Online survey.	Brief Symptom Inventory (BSI- 18), Checklist development (COPE: COVID-19 & Perinatal Experiences)	depression, anxiety & global psychological distress, sleep, stress	behavioural coping strategies: passive coping strategies (screen time, social media, and intake of comfort foods) and active coping strategies (social support, and self-care)	Phenotypes with high levels of passive coping strategies were associated with elevated symptoms of depression, anxiety, and global psychological distress, as well as worsening stress and energy levels, relative to other coping phenotypes. In contrast, phenotypes with high levels of active coping strategies were associated with greater resiliency relative to other phenotypes	Pregnant women Active-coping profile – were more likely to have fewer children, and were less likely to identify as Black or Asian High-coping profile was also more likely to have fewer children and greater educational attainment. Passive-coping profiles were marginally more likely to have greater educational attainment, and were less likely to identify as Black. Postpartum women- postpartum women in the passive-coping profile were more likely to have greater educational attainment to be younger in age, and to identify as Black. Active-coping and high-coping profiles were more likely to have greater educational attainment.
3.	Kishore et al., (2018) (21)	India, 589 women who were between 6 and 20 weeks of gestation at the time of first contact, Non COVID-19(October 2014 and November 2015), large scale longitudinal cohort study.	Social Readjustment Rating Scale; Edinburgh Postnatal Depression Scale (EPDS) and Connor–Davidson Resilience Scale-10	Depression	Social support	Pregnant women who experienced life events may experience depression during the 1st trimester of pregnancy, but the effect could possibly be reduced by enhancing the social support not by resilience. Study indicates that resilience is weakly associated with perinatal depression suggesting that resilience alone is not sufficient as a factor to protect women from depression during pregnancy.	Depression: 6.5% had significantly higher number of life events, lower resilience scores & lower perceived social support as compared to those who have no depression. Life events predicted depression during pregnancy; however, the relationship was moderated by social support but not by resilience. Life events associated depression includes marital life (events, health, social and financial etc.).
4.	Patil et al., 2021 (32)	India, (n = 458), Pregnant women were recruited into the PRAMMS cohort between October 2014 and November 2015, consecutive sampling.	Edinburgh Postnatal Depression Scale (EPDS, Connor-Davidson Resilience Scale-10, Zimet’s Multidimensional Scale of Perceived Social Support.	lifetime trauma and postpartum depression	role of resilience and social support	Social support negatively mediated the association between lifetime trauma and postpartum depressive symptoms. However, resilience was not a statistically significant mediator.	Lifetime trauma was associated with postpartum depressive symptoms. Intrinsic factors such as resilience was not found to be a significant mediator of the association, suggesting factors external to the individual, such as social support are more influential in preventing postpartum depression.
5.	Di Paolo et al., 2022 (33)	Australia, COVID-19 pandemic, August 2nd - November 29th, 2020, sample = 419 pregnant women & two months postpartum, longitudinal cohort (The Birth in the Time of COVID (BITTOC)), online using snowball method	Depression, Anxiety and Stress Scales (DASS-21), BITTOC Assessment of Stress due to COVID-19 (BASC) Scale, Subjective Distress 200 scale, Brief Resilience Scale (BRS), Intolerance of Uncertainty Scale (IUS).	objective hardship and subjective distress, mental health,	Resilience, tolerance of uncertainty, and a positive cognitive appraisal.	Women with low/neutral resilience, or low/moderate tolerance of uncertainty, or a negative cognitive appraisal, greater objective hardship predicted higher postpartum anxiety. Women with high resilience, or high tolerance of uncertainty, or neutral/positive cognitive appraisal, there was no association. Only a neutral/positive cognitive appraisal significantly buffered the effect of subjective distress on anxiety.	Psychological intervention, including targeting positive reappraisal and reframing with cognitive behavioral therapy, would not only be beneficial for the mother, but also for her unborn child as high levels of stress in pregnancy are associated with offspring neurodevelopment, cognitive development, and temperament. Women with high resilience tend to focus on the positives or seek social support, as opposed to negative coping styles, such as avoidance which has been associated with postpartum depression.
6.	Harville et al., 201 (34)	Southern Louisiana, USA, 222 pregnant & 292 postpartum women, January 2006 and May 2007, cross sectional	EPDS (Edinburgh Postnatal Depression Scale, 17-item inventory of PTSD-like symptoms, Support Behaviors Inventory,	Depression, PTSD	Resilience Social support	35%of pregnant and 34% of the postpartum women were resilient from depression, whereas 56% and 49% were resilient from PTSD.	Resilience was most likely among White women, older women, and women who had a partner. A greater experience of the storm, particularly injury/illness or danger, was associated with lower resilience. Many people are resilient after terrible events, and even the worst events sometimes have a positive side.
7.	Sexton et al., (2015) (35)	USA, (N=214), longitudinal study, postpartum women, Interview, study period not stated	Connor-Davidson Resilience Scale (CD-RISC) and Childhood Trauma Questionnaires (CTQ), Postpartum Depression Screening Scale (PDSS), National Women’s Study PTSD Module (NWSPTSD).	childhood history of maltreatment on posttraumatic stress disorder (PTSD), major depressive disorder (MDD),	Resilience, parental sense of mastery, and family functioning.	Resilience is associated with reduced psychopathology and improved wellbeing in all mothers. It further serves as a buffer against psychiatric symptoms following childhood trauma. lower resilience and greater maltreatment severity were both associated with increased rates of PTSD. Resilience and the trauma were predictive of postpartum family functioning, though no moderating influence of resilience on childhood trauma was found.	In mothers without childhood maltreatment, PTSD was absent irrespective of resilience scores. However, for those with the highest quartile of trauma severity, 8% of those with highest resilience in contrast with 58% of those with lowest CD-RISC scores met PTSD diagnostic criteria. For those with highest resilience, no mothers met criteria for postpartum MDD, irrespective of childhood trauma,.
8.	Julian et al., 2021 (18)	USA, 233, prospective longitudinal study, pregnancy through postpartum, interviewer administered questionnaires,	stressful life events (SLEs), Pearlin Mastery Scale, Life Orientation Test (LOT-R), Daily Spiritual Experiences Scale (DSES), Edinburgh Postnatal Depression Scale (EPDS).	Stressful life events during pregnancy (41%), Early postpartum depression (10%)	mastery, dispositional optimism, and spirituality	Mastery and optimism predicted fewer symptoms of depression postpartum. Mastery moderated the association between stressful life events and symptoms of depression when controlling for previous psychiatric history.	Interventions focusing on promoting a woman’s sense of mastery and coping skills earlier in life as a means of preparing women for healthier lives later on, including during their pregnancies and after with special focus on racial (black), poorer clinical outcome, financial hardship and ethnic minority women.
9.	Ma et al., 2019 (36)	Shanghai, prospective cohort study, 2813 sample, study on pregnant mothers, April2016- Feb 2018, Self-completion of the questionnaires	Stress- The Life Event Scale for Pregnancy Women (LESPW), Self - Rating Anxiety Scale (SAS), Depression -The Center for Epidemiological Survey, Depression Scale (CES-D) -Resilience - resilience Scale for Adults (RSA)	Anxiety- 11.1% Depression - 10.3%	Family and society and the life is stable	Resilience was a protective factor both prenatal anxiety/depression	Residence area, years in Shanghai, age, education level, working status, and family income were associated with resilience level, which could provide evidence for future intervention studies and more factors should be considered in future research.
10.	Alves et al., 2023 (37)	Brazil, cohort study,383 pregnant women, March 2018 to March 2020, checklist	- Perceived Stress Scale, Resilience was assessed by the Wagnild and Young Resilience Scale	Perceived stress, resilience	Resilience	Pregnant women with low resilience scores had higher perceived stress scores.	Women with a low resilience score (RS < 125) were more likely from the Northeast region, adolescents, other than whites, did not study or work, had a low level of education, low family income and received public antenatal care.
11.	Di Paolo et al., 2022 (33)	- Saudi Arabia -1409 postpartum women - February & May of 2022 cross-sectional study - Social media platforms (Email, Twitter, and WhatsApp)	- Edinburgh Postnatal Depression Scale (EPDS), Sleep quality was assessed using the Pittsburgh Sleep Quality Index (PSQI), Brief Resilience Scale (BRS)	PPD 75% a risk of PPD, sleep problems, 97% reporting having sleep problems, and 36% being in the “ low resilience level”	psychological/psychosocial	poor sleep quality, and low resilience levels were at high risk of developing PPD	Not reported
12.	Kishore et al., 2018 (20)	Boston,Massachusetts, 30 pregnant women, USA, March 1 to October 31, 2014 - Cohort study Self-report and a retrospective chart review	Connor–Davidson Resilience Scale (CD-RISC 25) -Depression -Patient Health Questionnaire (PHQ-9)	Depression- 73% Resilience = 82%	having adequate financial resources -religious affiliation	Neither anxiety nor substance use was associated with resilience	Nulliparity associated with resilience. Median resilience scores were significantly lower among women with a history of depression (73.0 [IQR 66.0–81.0]) than among those without a history (85.0 [IQR 79.0–92.0]; P=0.007). A history of using medication for anxiety, depression, or insomnia before pregnancy was also associated with lower resilience (median). Higher resilience was associated with religious affiliation and having adequate financial resources.
13.	Kornfield et al., 2021 (38)	-USA - cohort study -833 pregnant -April–July 2020 -online survey	Edinburgh Postnatal Depression Scale (EPDS), Anxiety Disorder -(GAD-7), Brief Risk and Resilience Battery	Anxiety -125 Depression - 31%	- Emotion regulation, - Self-reliance, & non hostile relationships	Depression was negatively affect resilience.	Emotion control exhibits considerable protection against postpartum depression, and self-reliance and non-hostile interactions are resilience variables that appear protective for both postpartum depression and decreased mother-infant attachment.
14.	Abera et al., 2023 (39)	-Jimma, comparative cross-sectional study design, -166 pregnant women, -September to 30 November 2021, interview method	Perceived Stress Scale (PSS-10), Brief Resilience Scale (BRS)	-perceived stress was 89% in pregnant women - The proportion of low resilience was 46.7% for pregnant	-Social support	Perceived stress is higher and resilience is lower in pregnant women. Pregnancy was associated with increased stress score by 4.1 points, with reduced resilience by 3.3 points in a fully adjusted model	-Determinant of resilience household food insecurity, distress and increased physical activity -There is a need for more research into the different stress response mechanisms and stress biomarkers during pregnancy
15.	Salazar-Pousada et al., 2010 (40)	Ecuador, Case-control study, 1 February 2010 to 30 April 2010, Nulliparous women, 50 (125 subjects per group), Interviewer administered questionnaires.	Center for Epidemiologic Studies Short Depression Scale (CESD-10) & Wagnild and Young Resilience Scale (RS).	Depression (56.6%)	Resilience social support	Adolescents displayed a lower level of resilience when compared to young adult gravids.	Having an adolescent partner and a preterm delivery related to a higher risk for lower resilience. Social support should be provided throughout pregnancy in order to increase resilience in our adolescent population
16.	Olajubu et al., 2021 (41)	Nigeria,analytical cross-sectional study, 241 pregnant adolescent, Study period … structured questionnaire	Perceived Stress Scale, and Wagnild Young Resilience Scale	80.5% perceived pregnancy-related stress -77.2% had low level of resilience	Support	Inverse relationship was found between perceived pregnancy-related stress and resilience	-older age groups and those who had support associated with resilience.
17.	Luo et al., 2021 (42)	China, February 28, 2020 to April 26, 2020, a sample of 2,116 pregnant women, online self-reporting, Latent profile analysis???	-Perceived Stress Scale (PSS-10), Connor-Davidson resilience scale (CD-RISC), Generalized Anxiety Disorder scale (GAD-7	Perceived stress	Person-centered approach, and provided initial evidence stress interventions	Resilience reduces their anxiety. Perceived stress profiles: adaptive: (33.7% of the sample), resistant (44.6%), insensitive (19.1%), and sensitive (2.6%)	The effects of the differences between adaptive/insensitive and resistant profiles on anxiety were partially mediated by resilience.
18.	Zhang et al., 2020 (43)	China, cross sectional study, 605 pregnant women, July 2018 to July 2019, online questionaries’	Perceived Stress Scale (PSS), Ego Resilience Scale, Center for Epidemiologic Studies Depression Scale (CES-D)	prenatal depression-28.4%	Woman’s harmonious relationship with her own mother (family relationship)	Prenatal depression and resilience have strong negative relationship.	Pregnant woman’s harmonious relationship with her own mother and resilience could relieve the negative impacts of pregnancy that can lead to prenatal depression.
19.	Tuxunjiang et al., 2022 (44)	China, 750 pregnant women December 2020 to May 2021, distribute questionnaires, survey	Pregnancy Pressure Scale, PPS, Generalized Anxiety Disorder-7, (Connor Davidson resilience scale (CD-RISC))	16.2% had moderate or greater pregnancy stress & 32.1% had higher mental resilience score	Tenacity Strength Optimism	-Pregnancy stress negatively affected resilience - Resilience also negatively affected prenatal anxiety. The mediating effect value of resilience was 8.3%.	Pregnancy stress, mental resilience, and prenatal anxiety were significantly correlated, and mental resilience played a partial mediating role in the influence of pregnancy stress on prenatal anxiety.
20.	Li et al., 2016 (45)	China,230 pregnant women,	Pittsburgh Sleep Quality Index (PSQI), Pregnancy Stress Rating Scale (PSRS), Connor-Davidson Resilience Scale (CD-RISC-10).	Sleep Stress	Resilience	Resilience mediated the relationship between prenatal maternal stress and sleep quality. Higher prenatal maternal stress was related to lower resilience and worse sleep quality, while higher resilience was related to better sleep quality).	Risk factor for disturbed sleep was pregnancy-specific stress Resilience was positively associated with sleep quality.
21.	Mei et al., 2022 (46)	China, 1,060 Chinese pregnant women, cohort study, January 2022 and April 2022. interview	The Childbirth Attitudes Questionnaire (CAQ), The Perceived Social Support Scale (PSSS), Connor-Davidson Resilience Scale (CD-RISC), General Self-efficacy Scale (GSES)	-Resilience could explain 41.6% and fear of childbirth 33.1% of psychological distress	-relieving psychological distress level	high resilience associated with low fear of childbirth	Pregnant women with high resilience-low fear of childbirth had significantly lower levels of psychological distress than those with low resilience-high fear of childbirth. The indirect effect of fear of childbirth on psychological distress through resilience was significantly. The interactions between fear of childbirth and adverse childhood experiences and between resilience and adverse childhood experiences were significant
22.	Sójta et al., 2023 (47)	Polish, 17 February to 13 October 2021, longitudinal study, using social media, 122 perinatal women.	Edinburgh Postnatal Depression Scale (EPDS), Beck Depression Inventory (BDI - 2), Resilience Measure Questionnaire (KOP26)	26.2% had depressive symptom	Psychoeducation	Low resilience was significantly associated with depressive symptoms and anxiety related to childbirth.	Findings highlight the importance of considering resilience as an important factor in understanding and managing perinatal depression and may have implications for the development of targeted interventions.
23.	Huang et al., 2022 (48)	China, Cross sectional study, 579 pregnant women, December 2021 to April 2022, self-filled questionnaires	Chinese Pregnancy-related Anxiety scale, Connor-Davidson Resilience Scale, Multidimensional Scale of Perceived Social Support	Pregnancy related anxiety 41.4%	Family function and perceived social support	Resilience and perceived pregnancy related anxiety have inverse relationship	- perceived social support and family function factors for resilience.
Mixed							
24.	Carlin et al., 2021 (49)	Australia, perinatal women, COVID-19 Quantitative: 174 (31 pregnant and 143 were postpartum- up to one-year post-birth) Qualitative: 14 interviews using Purposive sampling Qualitative: online using Semi-structured interviews Quantitative: online developed non validated tool	Mental Health Continuum—Short Form (MHC-SF), mindfulness Attention Awareness Scale (MAAS), Self-Compassion Short Form Scale (SCS), Perceived Stress Scale (PSS).	perceived stress & wellbeing	mindfulness and self-compassion	The relationship between mental health and resilience revealed positive association. resilience traits and positive mindsets may be protective against psychological distress for the mother and her child	Meditation-based or similar training for expectant women might help support resilience them during times of crisis, such as a pandemic.
25.	Shadowen et al., 2022 (50)	sample of Aboriginal women’s, Australia pregnant (more than 6 weeks gestation) & had a child aged between 7 days and 12 months, Non-COVID-19 (2013–2014), qualitative - Sample (91) using retrospective survey. Quantitative- not explained	Kimberley Mum’s Mood Scale (KMMS)	Depression and/or anxiety (25%)	Family-based support (mothers followed by sisters), self-care	Protective –family, healthy lifestyle,emotional self-regulation, good childhood experience Risky - loss and grief (managed by family support), IPV.	family as stress, lack of emotional regulation/self esteem and intimate partner violence were individual significantly associated with higher KMMS risk and having clinical depression and/or anxiety. Disclosure of IPV, experienced childhood adversities, was significantly associated with a diagnosis of a depression and anxiety.
26.	Ma et al., 2019 (36)	USA, women during 2–6 months, postpartum, Quantitative=medical record review and an electronic survey & Semi-structured individual interviews for qualitative	Quantitative= Brief Assessment of Recovery Capital (BARC-10), qualitative= interview guide based on the (Substance Abuse and Mental Health Services Administration (SAMHSA)’s recovery framework served).	Opioid Use Disorder	Recovery	Consistent with SAMHSA’s framework, our participants’ Substance use management improved in recovery and enabled recovery progress.	Recovery goals included = no use of drugs or alcohol (62.5%), being a better partner/spouse (87.5%), and improving finances (87.5%). Qualitative = recovery as transformative, building resilience, and transforming one’s health, relationships, and environment through recovery.
27.	Huang et al., 2022 (51)	USA, n =524, cross-sectional observational study survey, pregnant and postpartum (up to 6 months post-delivery) women. During COVID-19 pandemic from April–June, 2020, Online data collection method,	Brief Symptom Inventory-18 (BSI), post-traumatic stress disorder (PTSD) checklist for DSM-5 (PCL-5), Connor-Davidson Resilience Scale (CD-RISC 2), Coronavirus Perinatal Experiences Impact Survey (COPE-IS).	depression, Anxiety and post-traumatic stress disorder.	Social support and self-care	Women with family and job concerns and low resilience/adaptability scores seem to be at high risk of psychological sequelae. Use of social media is thought to improve social connectedness, our results indicate that increased media consumption is related to increased anxiety symptoms.	Determinants of outcome variable: Quantitative: job insecurity, family concerns, eating comfort foods, resilience/adaptability score, sleep, and use of social and news media. Qualitative themes pervasive uncertainty and anxiety; grief about losses; gratitude for shifting priorities; and use of self-care methods including changing media use. Commonly utilized self-care practices: changing their relationship with technology to maintain support with friends, family members, and healthcare providers) and engaging in self-care activities (e.g., exercise, time outdoors, eating well, prayer)).
28.	Abera et al., 2023 (39)	USA, 31 pregnant and postpartum women, During (COVID-19) pandemic, online survey,	Patient Health Questionnaire–2 (PHQ-2), Generalized Anxiety Disorder–7 (GAD-7),Brief Resilience Scale (BRS), Warwick-Edinburgh Mental Wellbeing Scale (WEMWBS),Loneliness Scale,	12% of the high depressive symptom and 60% reported moderate or severe anxiety. 40%of the sample reported being lonely	Self-care Being outdoors, gratitude, adhering to routines	Various resilience mechanisms found to have positive effect on the mental health condition.	Qualitative data suggested that social support, and specifically partner and emotional support, gratitude and optimism and the management or shifting of expectations were significant protective factors for pregnant and postpartum women, particularly during exposure to significant environmental stressors. Quantitative: use of virtual communication platforms, engaging in self-care behaviors, partner emotional support, being outdoors, gratitude, and adhering to structures and routines.

Twenty-three of the studies were quantitative observational, and five were mixed-methods research. Nine of the observational quantitative studies were carried out during the COVID-19 era, and twenty-two studies equally shared eleven cross-sectional and longitudinal cohorts amongst them. A total of 17,453 sample sizes were included, with the minimum and maximum sample sizes in the current review evidenced in the USA ranging from 30 to 4412 (31, 52).

Almost all the studies included in the current review used standard and cross-culturally validated instruments to assess mental health outcomes. Accordingly, the Connor-Davidson Resilience Scale-10 and 25 items (CD-RISC) were the most commonly used instruments in more than 45% of the retrieved reviews (13 out of 28 studies) (21, 30, 32, 35, 40, 42, 43, 51–56) for measuring resilience, followed by the Brief Resilience Scale (BRS) (33, 39, 57, 58) and the Wagnild Young Resilience Scale (37, 40, 41). The other instruments used for the assessment of resilience include the Resilience Measure Questionnaire (KOP26), the Ego Resilience Scale, the Brief Risk and Resilience Battery, and the Resilience Scale for Adults (RSA). Even if the study period was undertaken during the perinatal period from conception to the postnatal period, eleven studies were exclusively undertaken during pregnancy, while the rest of the studies were done during both the pregnancy and postpartum periods. Considering the mental health conditions examined for their association with resilience, depression, and anxiety take a leading role in most of the studies, followed by perceived stress.

The Edinburgh Postnatal Depression Scale (EPDS) was commonly used for assessing postpartum depression in the current review (8 of the 28 studies) (18, 21, 30, 32, 34, 38, 57, 59), followed by the Epidemiologic Studies Depression Scale (CES-D) in about four studies (3/28) (36, 40, 43), and Patient Health Questionnaire-2 (PHQ-9)(2/28) (52, 58) Moreover, the Beck Depression Inventory (BDI-2) (59) and the Depression, Anxiety, and Stress Scales (DASS-21) (33) were also employed to measure depression in the retrieved reviews. Of the 28 studies, eight were completed between 2009 and 2019 (21, 34–36, 38, 40, 52, 54), and twenty were undertaken between 2021 and 2023 (18, 30, 31, 33, 37, 39, 41–43, 49–51, 53–60) **Table 1**.

Regarding risk of bias, we utilized the Joanna Briggs Institute (JBI) criteria to assess the quality of the quantitative research, and we discovered that twenty one of the studies had low risk bias (18, 21, 30–37, 39–43, 51–54, 57, 59), two had medium risk (38, 57), and no study reported had high risk. The MMAT was another method used to assess the quality of the mixed research. According to previously published systematic literature reviews as well as recommended practices for using the MMAT to communicate the quality of the study, overall quality rating scores have been given. Accordingly, four studies, each two of them scored 60% and 80%, respectively, and one study scored 100% **Supplementary File 1**.

### Mental health outcome of study respondents

According to the studies reviewed in this review, there are differences in the types and degree to which different mental health problems are associated with resilience. This includes depression, which is the most commonly studied common mental health condition in more than 50% of the studies (16/28 studies) (18, 21, 30–32, 34–36, 38, 40, 43, 49, 50, 52, 56, 58, 59), followed by anxiety disorders (8/28 studies) (30, 31, 36, 38, 49, 51, 56, 58). The third commonly investigated mental health condition is perceived pregnancy stress (7/28 studies) (18, 37, 39, 41, 42, 53, 60). Post-traumatic stress disorder was another condition commonly reported (3/28) (34, 35, 56), followed by psychological distress (31, 54) and sleep disorders (31, 55, 57). Besides, opioid use disorders, lifetime trauma, childhood maltreatment and fear of childbirth were the least reported in the current review (32, 35, 50, 55). The magnitude of depression has been reported in about 13 studies in various settings, ranging from 6.5% in India (21) to 75% in Saudi Arabia (57), both for antenatal and postnatal depression, and the report seems lower as to anxiety disorders comparatively (ranging from 11.1% to 60%). Additionally, more perceived pregnancy stress was reported than any other condition, ranging from 16.2% in China to 89% in Ethiopia.

### Association of mental health with resilience

Studies have shown that resilience can protect us from psychological disorders, and this could take different forms, such as directly reducing the impacts of the condition or offsetting determinants that increase the risk of mental health conditions. Particularly during the perianal period, numerous circumstances affect the resilience of individuals living with mental health conditions, which might vary depending on the perinatal period (pregnancy and postnatal), parity, individual capability, sociocultural, environmental, and other significant attributes.

Accordingly, the current review highlighted the highest resilience scores for pregnancy compared to the postpartum period, specific to psychological disorders such as depression and PTSD. Resilience was found to decrease 34% of depression and 49% of post-traumatic stress disorder in the postpartum period, whereas 35% of depression and 56% of post-traumatic stress disorder occurred during pregnancy (33). In line with the current findings, a lower score of resilience was found to have an association with the highest score of depression and PTSD in the presence of a maternal history of childhood maltreatment (35). Psychological distress was the other mental health outcome and was 41.6% explained by resilience (55). In our review, the lowest score of resilience among pregnant women ranges from 36% in Saudi Arabia (57) to 77.2% in Nigeria (40), with the highest score evidenced among nulliparous women compared to those in the pregnant and postnatal periods (52). In sum, studies in the current review reported a higher resilience score with reduced mental health outcomes, including depression and anxiety (18, 21, 30, 31, 33, 34, 36, 38, 43, 51, 56, 58, 59), PTSD (34, 35, 56), stress (18, 37, 39, 42, 53), psychological distress (31), and sleep disturbance (54, 57). In contrast to this, study from Massachusetts, USA, among pregnant women show neither anxiety nor substance use was associated with resilience (52), and similarly, resilience did not significantly mediate the association of lifetime trauma and postpartum depression (32). This might be explained from dose response relationship in which low exposure to adversity help them to learn on how to cope with or become resilient to negative experiences and thereby improve their mental health. Conversely, individuals who face hardships on a regular basis could give up on hope for better results, while those who never face difficulties are unable to acquire coping mechanisms, increasing their chance of developing mental health conditions. Moreover, a study found that the absence of a relationship between anxiety and resilience might be related to the study’s use of self-reported anxiety (20) rather than a validated measure (61).

### Mechanism of resilience and mental health outcome

Physiological, neurobehavioral, and psychological factors are highlighted in models of psychological resilience as important contributors to safeguarding resilience. Psychological factors, including optimism, self-efficacy, high IQ, and the application of responsive emotional regulation techniques, have all been demonstrated in the earlier report to make a positive contribution to resilience (62–65). Resilience is a risk factor for some clinical diseases, such as suicide, as well as a protective factor against the onset of mental disorders. The available data from research studies indicates that resilience is a controllable factor, which creates opportunities for several novel psychosocial and biological therapies.Likewise, numerous mechanisms or techniques of resilience from perinatal mental health outcomes have been identified in the current review, including financial or psychological support (30, 52, 55, 57), behavioral coping strategies (passive coping strategies (screen time, social media, and intake of comfort foods), and active coping strategies (social support) (21, 31, 32, 34, 36, 39–41, 51, 56, 57) and self-care). A study revealed women with active coping skills and high coping profiles were more likely to have greater educational attainment compared to those with passive coping (31). According to a study, women who practice active coping strategies, including situational adjustment, accommodation, and symptom reduction, are more likely to maintain their social networks, take care of themselves, stay away from harmful material that could affect their health, and spend less time vegging out. Passive coping pregnant women avoided social interaction, concealed their feelings from others, and seldom employed self-stimulation or diversion during stressful situations such as COVID-19. Moreover, avoidance was the least common prenatal coping strategy, while prayer was the most commonly used, and higher levels of uncertainty were associated with lower emotional well-being, less positive interpretation, less social support, and greater avoidance during pregnancy (31, 66). A study revealed that pregnant women benefit intellectually and emotionally from psychological support, which also eases their transition to parenting and they may feel more stress and worry related to childbirth if they don’t have psychological support (67).

In the context of a secure existence with family or society, low resilience was substantially linked with reduced depressive symptoms and anxiety connected to childbirth. Also, the provision of psycho-education strengthens the negative impact of resilience on mental health conditions among pregnant and postpartum women (36, 59). Depending on the social context in which it was placed, a particular stressor may intensify or lessen, and it may also be viewed as posing varying degrees of perceived stress or requiring varying levels of resilience. In addition, being able to tolerate uncertainty and having a positive cognitive appraisal (33) were also protective measures for explaining resilience during the perinatal period. Consequently, it was discovered that women with lower resilience, lower uncertainty tolerance, a negative cognitive evaluation, and greater objective suffering were more likely to have postpartum anxiety. Only a neutral or positive cognitive appraisal was able to considerably lessen the impact of subjective discomfort on anxiety. Women with high resilience are more likely to turn to positive reinforcement or seek out social support than to use negative coping methods linked to depression after childbirth. A neutral or positive cognitive appraisal significantly mitigated the influence of subjective distress on anxiety (33).

Implementation of person-centered strategies including mindfulness and self-compassion (60), regulating emotions (58), self-reliance and non-hostile interactions (38), tenacity and strength (53), and offering early evidence stress interventions (42), has been demonstrated to influence resilience in pregnant women. This supported the idea that resilience shows a robust link by playing a mediating role in the influence of pregnancy stress on prenatal anxiety. Positive attitudes and resilience attributes can safeguard both the mother and the child from psychological suffering (42, 53).

In the context of severe psychosocial stressors or crises such as the COVID-19 pandemic, women reported the use of online communication platforms, engaging in self-care behaviors (49, 56, 58)(e.g., adequate sleep, physical activity, and healthy eating), seeking partner emotional support, being outdoors, practicing gratitude, and adhering to structures and routines shown to enhance resilience. Furthermore, a study conducted in the USA examined the effectiveness of a recovery-oriented intervention among postpartum women with opioid use disorder utilizing the Substance Abuse and Mental Health Service Administration (SAMHSA) framework. It has been demonstrated to have a positive impact on resilience and facilitate recovery (50).

Numerous studies have provided explanations for coping strategies that lessen the adverse effects of mental health disorders on prenatal populations, one of which is social support.

Consequently, a study by Prabha S et al. found that social support, rather than resilience, mediated the link between life events and depression during the first trimester of pregnancy. This demonstrates that resilience alone cannot avoid depression (21). Likewise, the scenario also applies to the postpartum phase, where resilience was unable to statistically buffer the correlation between lifetime trauma and postpartum depressive symptoms, and social support negatively mediated the relationship. It is important to offer social support to expectant mothers in order to build resilience in our adolescent population (40, 41). Social support and resilience demonstrated an inverse association with anxiety (51) and will be improved by adjusting our relationship with technology, particularly during the COVID-19 pandemic (56).

Another resilience approach that affected the mental health outcomes of pregnant and postpartum women was practicing self-care behaviors, such as getting enough sleep, following routines, being grateful, exercising, eating healthily, spending time outside, and praying (49, 56, 58). Given this, women who struggle with family and work-related issues and who score poorly on resilience and adaptation appear to be more vulnerable to psychological disorders.

Additionally, social media use is expected to enhance social connectivity, which in turn increases anxiety symptoms. Family functioning is the extent to which a family functions as a unit to manage circumstances, events, outside stimuli, or activities that produce stress. In the current review, family functioning (35, 51), specifically a woman’s positive relationship with her own mother (43), followed by a sister (49) was shown to inversely affect anxiety and depression and promote well-being among pregnant women. Resilience in mothers is linked to a decrease in psychopathology and an increase in general well-being. While there was no evidence of resilience having a moderating effect on childhood trauma, resilience and trauma were predictive of postpartum family functioning. It also acts as a protective barrier against mental health conditions that arise from childhood trauma (35).

It has been shown that spirituality and a variety of psychosocial resources, such as dispositional optimism, parental sense of mastery, self-esteem, gratitude, and forgiveness, positively correlate with resilience and affect the mental health outcomes of perinatal women. According to a study, among postpartum women, depression was predicted by optimism and mastery but not by spirituality. The only resilience resource that can moderate the association between the number of stressful life experiences and postpartum depression symptoms with a small effect size is mastery (18, 52, 53).

### Determinants of resilience among women in the perinatal period

Various factors influence perinatal women’s resilience in terms of mental health outcomes. Even the worst situations can occasionally have a silver lining, and many people bounce back from traumatic experiences with resilience. In the current review, resilience was associated with among older (34, 36, 37, 41), white (34), residence area (34, 36), educational level (37), employment status (36, 37, 56), northeast region (37), having an adolescent partner (37, 40) family income (36, 37), and receiving public antenatal care (37), household food-security, distress, and increased physical activity (39), and women having a partner while experiencing higher extreme weather events (storms), especially one involving threat, sickness, or trauma, was linked to poorer resilience (34, 36).

Compared to women without a history of depression, women with a history of depression had considerably lower median resilience ratings. Lower resilience was also linked to a prior history of using medication for depression, anxiety, or sleeplessness before becoming pregnant (median). Belonging to religion and having sufficient finances were connected with higher resilience (38).

Moreover, preterm delivery (40) and poor sleep quality (53) were related to a higher risk of lower resilience. Psychological distress was much lower in pregnant women with strong resilience and low fear of childbirth than in those with poor resilience and high fear of childbirth. Significant relationships were found between the fear of childbirth and negative childhood experiences, as well as resilience and negative childhood experiences (55).

Five of the retrieved reviews used mixed methods, and the qualitative results from these contributed to clarifying and amplifying the quantitative conclusions. As a result, the results of qualitative data suggests that positive family relationships suggested are protective factors and have the tendency to reduce anxiety and depression among perinatal women (49). Furthermore, Jacqueline A. et al.’s study, 2021 (60), conducted during the COVID-19 pandemic, examined resilience in prenatal mothers into four main qualitative themes, which are as follows: The first theme clarified a rise in psychological distress (stress, anxiety, and/or depression), especially during the period of acute constraint and family absence. The absence of in-person services, uncertainty about where to get them, and lack of information about hospital admission restrictions fall under theme two. Separation from friends and family, a greater need for assistance and guidance, and favorable outcomes (such as time-bound and flexible work schedules) fall under theme number four. In sum, women stated that throughout this period of their lives, support from peers, family, and friends, getting adequate information (58), being thankful for changing priorities, and using self-care techniques (56), were found crucial in helping them become resilient in the face of stressors like COVID-19 (49), which also explains the role of recovery in building resilience among substance users (50).

## Discussion

Over the past few years, policymakers, medical professionals, and researchers have shown an increased interest in the concept of resilience. This interest stems primarily from the potential benefits of resilience for happiness, health, and quality of life (68). Earlier research (36, 68–71) focused on the rare trajectories of the presence of difficulties or trauma, such as intimate partner violence or adolescent pregnancy, for the investigation of resilience during the perinatal period.

Furthermore, previous reviews on perinatal resilience-focused primarily on explaining how women’s resilience has been conceived and defined in scientific studies (72), while the current review aims at the evidence that connects resilience to common mental health outcomes like depression, anxiety, suicide, stress, sleep quality, substance abuse, and PTSD.

Comprehending the risk and promoting features of resilience provides many stakeholders with an opportunity to guarantee the well-being of mothers and children. In addition, the review investigated the specific resilience mechanisms used to mitigate the effects of mental health and related variables.

The current review conceptualizes resilience integration for evaluating its impact on perinatal women’s mental health outcomes, and nearly all of the studies (26/28) consider resilience to have a negative impact on mental health conditions and to promote well-being, regardless of the degree of association. However, the studies from Massachusetts, the USA, and India, where anxiety, substance use, and lifetime trauma lack associations with resilience, did not support this finding (32, 41). In other ways, studies among abstinent individuals with substance use disorder show resilient people disclose more about themselves, easily recover (50), and are less likely to relapse into alcohol use (73, 74).

The associations between mood disorders, trauma-related disorders, and resilience have been investigated in our reviews. Subsequently, higher scores of depression were shown to have an association with lower resilience scores. Resilience was found to decrease depression and post-traumatic stress disorder during the postpartum and pregnancy periods by 34% and 49% and 35% and 56%, respectively (34).

Moreover, resilience explained psychological distress by about 41.6% (55) and negatively affected other mental health disorders, including stress, sleep, and PTSD. This could be because psychological resilience enables individuals to build strong coping mechanisms, seek assistance when necessary, and apply their abilities to overcome obstacles in their mental health conditions. Besides, as a protective characteristic, resilience has been demonstrated to substantially decrease the correlation between risky exposure in life and depression, thereby functioning as a safeguard against adverse consequences including anxiety, depression, and post-traumatic stress disorder (75).

Family functions and social support are several instances of resilience mechanisms or coping strategies that have been invoked to explain mental health among pregnant women. As a result, previous reports indicate that women with depression and anxiety during the perinatal period received poorer social support (76), despite the fact that most of the retrieved reviews tested the provision of this support to buffer perinatal maternal adaptation to mental health outcomes and improve well-being. In other ways, Sexton et al. (35) hypothesized that the key components of lowering psychological distress are that resilient people are more likely to have supportive peers and to share their opinions.

It has also been demonstrated that family functioning negatively affects depression and anxiety while enhancing pregnant women’s well-being. Previous literature supported these findings (77, 78). This may result from the beneficial influence of fathers on their offspring as well as the cortisol responsiveness of newborns after a stressful event, which is partly mediated by a lower risk of antenatal depression and more optimistic mother-infant interactions (79).

In the current review, mastery was one of the resilience resources that have been shown to be essential for controlling the association between the number of stressful life events and postpartum depression symptoms. Women who experience a high sense of mastery are less vulnerable to the psychological impacts of stress because they are more likely to believe they can manage or control life’s obstacles (80).

Several factors impact the resilience of women in the perinatal period. For instance, older age was positively associated with resilience. According to particular situations and contexts, adaptive behavior rather than necessarily a progressively continuous quality might explain resilient behavior among older adults (81, 82). Lower family income and unemployed status among perinatal women were less likely to become resilient in response to mental health suffering compared to their counterparts. Employment is one significant factor in fostering mental wellness. It has a significant role in determining identity and self-worth. It can offer chances for relationships as well as a sense of fulfillment, and in most cases, their primary source of income comes from their job (83, 84). Therefore, increasing standards for employment and enhancing working conditions may help to reduce health inequalities and enhance wellbeing.

## Limitations of the study

This review included all articles regardless of time and geographical boundaries, addressing all quantitative and qualitative studies. Thus, it could be generalizable for the global population. However, the study excluded articles published in languages other than English, and data synthesis was not done, so the impact of heterogeneity, publication bias, and study variability were not assessed. Thus, this issue and some methodological issues, including study design shortcomings and sample size issues of the included studies, should be carefully considered while interpreting the findings.

## Conclusion

According to this systematic review, depression, anxiety, and post-traumatic stress disorders were found to be the most common, but not the only, mental health disorders during the perinatal period. The review found that higher maternal resilience during perinatal periods reduces mental health disorders. It was also found that pregnant women were more resilient to mental health disorders than postpartum women. Regarding the mechanisms of resilience to mental health disorders during the perinatal period, tolerating uncertainty and a positive cognitive appraisal, women’s self-behaviour and family functioning, and protective psychosocial resources such as dispositional optimism, parental sense of mastery, self-esteem, gratitude, and forgiveness were found to be the most common mechanisms of resilience. Older age, residence area, educational level, employment status, having an adolescent partner, family income, distress, and increased physical activity were found to be detrimental factors of resilience.

### Implication for practice

Noting that women’s resilience is an important tool to prevent perinatal mental health disorders, maternal healthcare providers need to counsel perinatal women on resilience-boosting mechanisms, such as applying self-behaviour and having social support or close family relationships. The current review took into consideration the need for healthcare providers to implement perinatal programs that aim to promote resilience by enhancing existing preventative measures and taking into account resilience mechanisms like active coping skills, self-care behaviors, social support, and cohesion. The integration of capacity building initiatives within current healthcare institutions could not only assist perinatal mothers, but also healthcare provider’s ability to provide quality services. Since high-level perinatal mental health conditions impair the biological and psychosocial development of the offspring, the mother and her unborn child would benefit more from positive reflection and protective psychosocial resources (85–89). We think it is also better to counsel the woman’s companion or partner to give strong support to the woman in each of the perinatal periods. Moreover, the concerned bodies need to incorporate counselling on resilience-boosting mechanisms into the antenatal and postpartum counselling packages.

## Data availability statement

The original contributions presented in the study are included in the article/**Supplementary Material**. Further inquiries can be directed to the corresponding authors.

## Author contributions

MJ: Writing – original draft, Writing – review & editing. SA: Conceptualization, Data curation, Investigation, Methodology, Writing – review & editing. ZA: Conceptualization, Investigation, Methodology, Writing – original draft, Writing – review & editing. GT: Data curation, Investigation, Methodology, Supervision, Writing – review & editing. YW: Conceptualization, Data curation, Investigation, Project administration, Writing – review & editing. AD: Conceptualization, Data curation, Investigation, Software, Supervision, Writing – original draft. MH: Conceptualization, Investigation, Methodology, Project administration, Writing – review & editing. LW: Conceptualization, Investigation, Methodology, Writing – original draft. WG: Writing – original draft.
