# Supplementary material for: Resilience and mental health among perinatal women: a systematic review
Source: Front Psychiatry. 2024 Jul 22;15:1373083. doi: 10.3389/fpsyt.2024.1373083 (PMC11298415; doi:10.3389/fpsyt.2024.1373083)
Supplement: Supplementary file 1 [file Table_1.docx]

**Supplementary file 1. Quality assessment of quantitative studies using JBI**

| Author & publication year | Was the sample frame appropriate to address the target population? | Were study participants sampled in an appropriate  way? | Was the sample size adequate? | Were the study subjects and the setting described in detail? | Was the data analysis conducted with sufficient coverage of the idenfied sample? | Were valid methods used for the identification of the condition? | Was the condition measured in a standard, reliable  way for all participants? | Was there appropriate statistical analysis? | Was the response rate adequate, and if not, was he low response rate managed appropriately? | Overall quality score |
| --- | --- | --- | --- | --- | --- | --- | --- | --- | --- | --- |
| Daniel Maria et al., 2021 | YES | NO | YES | YES | YES | NO | YES | YES | YES | 7 |
| Denise M et al.,2022 | YES | NO | YES | YES | NO | YES | YES | YES | YES | 7 |
| Prabha S et al.,2018 | YES | YES | YES | YES | YES | YES | YES | YES | NO | 8 |
| Veena A et al., 2021 | YES | YES | NO | YES | NO | YES | YES | YES | NO | 7 |
| Suzanne K et al.,2022 | YES | YES | YES | YES | YES | YES | NO | YES | YES | 8 |
| Emily H et al 2009 | YES | YES | YES | YES | YES | YES | YES | YES | YES | 9 |
| Maria M et al 2014 | YES | YES | YES | YES | YES | YES | NO | YES | YES | 8 |
| Melissa J et al 2021 | YES | YES | YES | YES | YES | YES | YES | YES | YES | 9 |
| Shi H et al., 2019 | YES | YES | YES | YES | YES | YES | YES | YES | YES | 9 |
| Jose G et al.,2023 | YES | YES | YES | YES | YES | YES | NO | YES | NO | 7 |
| Baian A et al.,2023 | YES | NO | YES | YES | NO | NO | NO | YES | YES | 5 |
| Jennifer S et al., 2017 | YES | YES | NO | YES | YES | NO | YES | YES | YES | 7 |
| Sara L et al., 2012 | YES | YES | NO | YES | NO | YES | NO | YES | YES | 5 |
| Mubarak A et al.,2023 | YES | YES | YES | YES | YES | YES | YES | YES | YES | 9 |
| Faustino R et al 2010 | YES | YES | YES | YES | YES | YES | NO | YES | YES | 8 |
| Aanuoluwap O et al.,2021 | YES | YES | YES | YES | YES | NO | YES | YES | YES | 8 |
| Zhengkui L et al., 2021 | YES | NO | YES | YES | YES | YES | NO | YES | YES | 7 |
| Xiaoshi Y et al., ,2020 | YES | YES | YES | YES | YES | YES | NO | NO | YES | 7 |
| Ting J et al 2022 | YES | YES | YES | YES | YES | YES | NO | YES | YES | 8 |
| Ping Li et al.,2016 | YES | YES | NO | YES | YES | YES | NO | YES | YES | 7 |
| Youjin L et al., 2022 | YES | YES | YES | YES | YES | YES | YES | YES | YES | 9 |
| Oliwia G et al.,2023 | YES | NO | YES | YES | NO | NO | YES | YES | YES | 6 |
| Yumei Shi et al.,2022 | YES | NO | YES | YES | YES | YES | NO | YES | YES | 7 |

**NB**: Low risk bias (20-50%), medium risk bias (50-80%), and high risk bias (80-100%)

**Supplementary 1.1** Mixed Methods quality appraisal criteria using Mixed Methods Appraisal Tool (MMAT; Hong et al., [Citation 2018](https://www.tandfonline.com/doi/full/10.1080/24732850.2021.2013364))

| **MMAT Criteria** | **Included studies** | | | | |
| --- | --- | --- | --- | --- | --- |
|  | Jacqueline A. et al 2021 | Emma C et al 2020 | Caroline Sh et al.,2022 | Patricia A et al 2021 | Charlotte V et al.,2020 |
| Clear rationale for mixed methods study? | NO | YES | YES | YES | YES |
| Components of the study effectively integrated? | YES | YES | YES | YES | YES |
| Outputs of the integration of qualitative and quantitative components adequately interpreted? | YES | NO | YES | YES | NO |
| Divergences and inconsistencies between quantitative and qualitative results adequately addressed? | NO | NO | YES | YES | YES |
| Different components of the study adhere to the quality criteria of each tradition of the methods involved? | YES | YES | NO | YES | YES |
| **Quality score** | 60% | 60% | 80% | 100% | 80% |
