# Supplementary material for: Resilience and mental health among perinatal women: a systematic review
Source: Front Psychiatry. 2024 Jul 22;15:1373083. doi: 10.3389/fpsyt.2024.1373083 (PMC11298415; doi:10.3389/fpsyt.2024.1373083)
Supplement: Supplementary file 2 [file Table_2.docx]

|  | | **Quantitative studies** | | | | | | | | |
| --- | --- | --- | --- | --- | --- | --- | --- | --- | --- | --- |
| S.N | | Author, year of publication | Study setting and period ,design , sample ,and Perinatal period and data collection methods | | Tool to assess resilience and mental health condition | Magnitude of mental health conditions | Resilience mechanism | | Association of resilience with mental health | Risk impacts or determinants/ future implication |
|  | | Daniel Maria et al., 2021 | Spain, during COVID-19, multi-centre cross-sectional survey, 514, During pregnancy , online self-administered questionnaire | | EPDS, State-Trait Anxiety Inventory and CDRIS-10 | Depression, (35.4%) & state anxiety (44.2%) | Financial support  Psychological support | | Resilience have moderate negative correlation with depression and anxiety. | Not reported |
|  | | Denise M et al.,2022 | USA, COVID-19 era, , sample of pregnant women (*N* = 2876) and postpartum women (*N* = 1536), Pregnant and postpartum period (first 12 months of infant life). Online survey. | | Brief Symptom Inventory (BSI- 18), Checklist development (COPE: COVID-19 & Perinatal Experiences) | depression, anxiety & global psychological distress, sleep, stress | behavioural coping strategies:  passive coping strategies (screen time, social media, and intake of comfort foods) and active coping strategies (social support, and self-care) | | Phenotypes with high levels of passive coping strategies were associated with elevated symptoms of depression, anxiety, and global psychological distress, as well as worsening stress and energy levels, relative to other coping phenotypes. In contrast, phenotypes with high levels of active coping strategies were associated with greater resiliency relative to other phenotypes | Pregnant women  Active-coping profile – were more likely to have fewer children, and were less likely to identify as Black or Asian  High-coping profile was also more likely to have fewer children and greater educational attainment.  Passive-coping profiles were marginally more likely to have greater educational attainment, and were less likely to identify as Black.  Postpartum women- postpartum women in the passive-coping profile were more likely to have greater educational attainment to be younger in age, and to identify as Black. Active-coping and high-coping profiles were more likely to have greater educational attainment. |
|  | | Prabha S et al.,2018 | India, 589 women who were between 6 and 20 weeks of gestation at the time of first contact, Non COVID-19(October 2014 and November 2015), large scale longitudinal cohort study. | | Social Readjustment Rating Scale; Edinburgh Postnatal Depression Scale (EPDS) and Connor–Davidson Resilience Scale-10 | Depression | Social support | | Pregnant women who experienced life events may experience depression during the 1^st^ trimester of pregnancy, but the effect could possibly be reduced by enhancing the social support not by resilience. Study indicates that resilience is weakly associated with perinatal depression suggesting that resilience alone is not sufficient as a factor to protect women from depression during pregnancy. | Depression: 6.5% had significantly higher number of life events, lower resilience scores & lower perceived social support as compared to those who have no depression.  Life events predicted depression during pregnancy; however, the relationship was moderated by social support but not by  resilience. Life events associated depression includes marital life (events, health, social and financial etc.). |
|  | | Veena A et al., 2021 | India, (n = 458), Pregnant women were recruited into the PRAMMS cohort between October 2014 and November 2015, consecutive sampling. | | Edinburgh Postnatal Depression Scale (EPDS, Connor-Davidson Resilience Scale-10, Zimet’s Multidimensional Scale of Perceived Social Support. | lifetime trauma and postpartum depression | role of resilience and social support | | Social support negatively mediated the association between lifetime trauma and postpartum depressive symptoms. However, resilience was not a statistically significant mediator. | Lifetime trauma was associated with postpartum depressive symptoms. Intrinsic factors such as resilience was not found to be a significant mediator of the association, suggesting factors external to the individual, such as social support are more influential in preventing postpartum depression. |
|  | | Suzanne K et al.,2022 | Australia, COVID-19 pandemic , August 2nd - November 29th, 2020, sample = 419 pregnant women & two months postpartum, longitudinal cohort (The Birth in the Time of COVID (BITTOC)) , online using snowball method | | Depression, Anxiety and Stress Scales (DASS-21), BITTOC Assessment of Stress due to COVID-19 (BASC) Scale, Subjective Distress 200 scale, Brief Resilience Scale (BRS), Intolerance of Uncertainty Scale (IUS). | objective hardship and subjective distress, mental health, | Resilience, tolerance of uncertainty, and a positive cognitive appraisal. | | Women with low/neutral resilience, or low/moderate tolerance of uncertainty, or a negative cognitive appraisal, greater objective hardship predicted higher postpartum anxiety. Women with high resilience, or high tolerance of uncertainty, or neutral/positive cognitive appraisal, there was no association. Only a neutral/positive cognitive appraisal significantly buffered the effect of subjective distress on anxiety. | Psychological intervention, including targeting positive reappraisal and reframing with cognitive behavioral therapy, would not only be beneficial for the mother, but also for her unborn child as high levels of stress in pregnancy are associated with offspring neurodevelopment, cognitive development, and temperament. Women with high resilience tend to focus on the positives or seek social support, as opposed to negative coping styles, such as avoidance which has been associated with postpartum depression. |
|  | | Emily H et al 2009 | Southern Louisiana , USA, 222 pregnant & 292 postpartum women, January 2006 and May 2007, cross sectional | | EPDS (Edinburgh Postnatal Depression Scale, 17-item inventory of PTSD-like symptoms, Support Behaviors Inventory, | Depression , PTSD | Resilience  Social support | | 35%of pregnant and 34% of the postpartum women were resilient from depression, whereas 56% and 49% were resilient from PTSD. | Resilience was most likely among White women, older women, and women who had a partner. A greater experience of the storm, particularly injury/illness or danger, was associated with lower resilience. Many people are resilient after terrible events, and even the worst events sometimes have a positive side. |
|  | | Maria M et al 2014 | USA, (N=214), longitudinal study, postpartum women, Interview , study period not stated | | Connor-Davidson Resilience Scale (CD-RISC) and Childhood Trauma Questionnaires (CTQ), Postpartum Depression Screening Scale (PDSS), National Women's Study PTSD Module (NWSPTSD). | childhood history of maltreatment on posttraumatic stress disorder (PTSD), major depressive disorder (MDD), | Resilience, parental sense of mastery, and family functioning. | | Resilience is associated with reduced psychopathology and improved wellbeing in all mothers. It further serves as a buffer against psychiatric symptoms following childhood trauma. lower resilience and greater maltreatment severity were both associated with increased rates of PTSD. Resilience and the trauma were predictive of postpartum family functioning, though no moderating influence of resilience on childhood trauma was found. | In mothers without childhood maltreatment, PTSD was absent irrespective of resilience scores. However, for those with the highest quartile of trauma severity, 8% of those with highest resilience in contrast with 58% of those with lowest CD-RISC scores met PTSD diagnostic criteria. For those with highest resilience, no mothers met criteria for postpartum MDD, irrespective of childhood trauma,. |
|  | | Melissa J et al 2021 | USA, 233, prospective longitudinal study, pregnancy through postpartum, interviewer administered questionnaires, | | stressful life events (SLEs), Pearlin Mastery Scale, Life Orientation Test (LOT-R), Daily Spiritual Experiences Scale (DSES), Edinburgh Postnatal Depression  Scale (EPDS). | Stressful life events during pregnancy (41%), Early postpartum depression  (10%) | mastery, dispositional optimism,  and spirituality | | Mastery and optimism predicted fewer symptoms of depression postpartum. Mastery moderated the association between stressful life events and symptoms of depression when controlling for previous psychiatric history. | Interventions focusing on promoting a woman’s sense of mastery and coping skills earlier in life as a means of preparing women for healthier lives later on, including during their pregnancies and after with special focus on racial (black), poorer clinical outcome, financial hardship and ethnic minority women. |
|  | | Shi H et al., 2019 | Shanghai, prospective cohort study, 2813 sample, study on pregnant mothers ,  April2016- Feb 2018, Self-completion of the questionnaires | | Stress- The Life Event Scale for Pregnancy Women (LESPW), Self - Rating Anxiety Scale (SAS), Depression -The Center for Epidemiological Survey, Depression Scale (CES-D)  -Resilience - resilience Scale for Adults (RSA) | Anxiety- 11.1%  Depression - 10.3% | Family and society and the life is stable | | Resilience was a protective factor both prenatal anxiety/depression | Residence area, years in Shanghai, age, education level, working status, and family income were associated with resilience level, which could provide evidence for future intervention studies and more factors should be considered in future research. |
|  | | Jose G et al.,2023 | Brazil, cohort study,383 pregnant women, March 2018 to March 2020, checklist | | - Perceived Stress Scale, Resilience was assessed by the Wagnild and Young Resilience Scale | Perceived stress, resilience | Resilience | | Pregnant women with low resilience scores had higher perceived stress scores. | Women with a low resilience score (RS < 125) were more likely from the Northeast region, adolescents, other than whites, did not study or work, had a low level of education, low family income and received public antenatal care. |
|  | | Baian A et al.,2023 | - Saudi Arabia -1409 postpartum women  - February & May of 2022 cross‑sectional study  - Social media platforms (Email, Twitter, and WhatsApp) | | - Edinburgh Postnatal Depression Scale (EPDS), Sleep quality was assessed using the Pittsburgh Sleep Quality Index (PSQI), Brief Resilience Scale (BRS) | PPD 75% a risk of PPD, sleep problems, 97% reporting having sleep problems, and 36% being in the “ low resilience level” | psychological/ psychosocial | | poor sleep quality, and low resilience levels were at high risk of developing PPD | Not reported |
|  | | Jennifer S et al., 2017 | Boston,Massachusetts, 30 pregnant women , USA, March 1 to October 31, 2014  - Cohort study  Self-report and a retrospective chart review | | Connor–Davidson Resilience Scale (CD-RISC 25)  -Depression -Patient Health Questionnaire (PHQ-9) | Depression- 73%  Resilience = 82% | having adequate financial resources  -religious affiliation | | Neither anxiety nor substance use was associated with resilience | Nulliparity associated with resilience. Median resilience scores were significantly lower among women with a history of depression (73.0 [IQR 66.0–81.0]) than among those without a history (85.0 [IQR 79.0–92.0]; *P=*0.007). A history of using medication for anxiety, depression, or insomnia before pregnancy was also associated with lower resilience (median). Higher resilience was associated with religious affiliation and having adequate financial resources. |
|  | | Sara L et al., 2012 | -USA  - cohort study  -833 pregnant  -April–July 2020  -online survey | | Edinburgh Postnatal Depression Scale (EPDS), Anxiety Disorder -(GAD-7),  Brief Risk and Resilience Battery | Anxiety -125  Depression - 31 % | - Emotion regulation,  - Self-reliance, & non hostile relationships | | Depression was negatively affect resilience. | Emotion control exhibits considerable protection against postpartum depression, and self-reliance and non-hostile interactions are resilience variables that appear protective for both postpartum depression and decreased mother-infant attachment. |
|  | | Mubarak A et al.,2023 | -Jimma, comparative cross-sectional study design ,  -166 pregnant women,  -September to 30 November 2021, interview method | | Perceived Stress Scale (PSS-10),  Brief Resilience Scale (BRS) | -perceived stress was 89% in pregnant women  - The proportion of low resilience was 46.7% for pregnant | -Social support | | Perceived stress is higher and resilience is lower in pregnant women. Pregnancy was associated with increased stress score by 4.1 points, with reduced resilience by 3.3 points in a fully adjusted model | -Determinant of resilience household food insecurity, distress and increased physical activity  -There is a need for more research into the different stress response mechanisms and stress biomarkers during pregnancy |
|  | | Faustino R et al 2010 | Ecuador, Case-control study, 1 February 2010 to 30 April 2010, Nulliparous women, 50 (125 subjects per group), Interviewer administered questionnaires. | | Center for Epidemiologic Studies Short Depression Scale (CESD-10) & Wagnild and Young Resilience Scale (RS). | Depression (56.6%) | Resilience  social support | | Adolescents displayed a lower level of resilience when compared to young adult gravids. | Having an adolescent partner and a preterm delivery related to a higher risk for lower resilience.  Social support should be provided throughout pregnancy in order to increase resilience in our adolescent population |
|  | | Aanuoluwap O et al.,2021 | Nigeria ,analytical cross-sectional study, 241 pregnant adolescent, Study period…structured questionnaire | | Perceived Stress Scale, and Wagnild Young Resilience Scale | 80.5% perceived pregnancy-related stress  -77.2% had low level of resilience | Support | | Inverse relationship was found between perceived pregnancy-related stress and resilience | -older age groups and those who had support associated with resilience. |
|  | | Zhengkui L et al., 2021 | China, February 28, 2020 to April 26, 2020, a sample of 2,116 pregnant women, online self-reporting, Latent profile analysis??? | | -Perceived Stress Scale (PSS-10), Connor-Davidson resilience scale (CD-RISC), Generalized Anxiety Disorder scale (GAD-7 | Perceived stress | Person-centered approach, and provided initial evidence stress interventions | | Resilience reduces their anxiety.  Perceived stress profiles: adaptive: (33.7% of the sample), resistant (44.6%), insensitive (19.1%), and sensitive (2.6%) | The effects of the differences between adaptive/insensitive and resistant profiles on anxiety were partially mediated by resilience. |
|  | | Xiaoshi Y et al., ,2020 | China, cross sectional study, 605 pregnant women, July 2018 to July 2019, online questionaries’ | | Perceived Stress Scale (PSS), Ego Resilience Scale, Center for Epidemiologic Studies Depression Scale (CES-D) | prenatal depression-28.4% | Woman’s harmonious relationship with her own mother (family relationship) | | Prenatal depression and resilience have strong negative relationship. | Pregnant woman’s harmonious relationship with her own mother and resilience could relieve the negative impacts of pregnancy that can lead to prenatal depression. |
|  | | Ting J et al 2022 | China, 750 pregnant women December 2020 to May 2021, distribute questionnaires, survey | | Pregnancy Pressure Scale, PPS, Generalized Anxiety Disorder-7, (Connor Davidson resilience scale (CD-RISC)) | 16.2% had moderate or greater pregnancy stress & 32.1% had higher mental resilience score | Tenacity  Strength  Optimism | | -Pregnancy stress negatively affected resilience  - Resilience also negatively affected prenatal anxiety.  The mediating effect value of resilience was 8.3%. | Pregnancy stress, mental resilience, and prenatal anxiety were significantly correlated, and mental resilience played a partial mediating role in the influence of pregnancy stress on prenatal anxiety. |
|  | | Ping Li et al.,2016 | China,230 pregnant women, | | Pittsburgh Sleep Quality Index (PSQI) , Pregnancy Stress Rating Scale (PSRS), Connor-Davidson Resilience Scale (CD-RISC-10). | Sleep  Stress | Resilience | | Resilience mediated the relationship between prenatal maternal stress and sleep quality. Higher prenatal maternal stress was related to lower resilience and worse sleep quality, while higher resilience was related to better sleep quality). | Risk factor for disturbed sleep was pregnancy-specific stress  Resilience was positively associated with sleep quality. |
|  | | Youjin L et al., 2022 | China, 1,060 Chinese pregnant women, cohort study, January 2022 and April 2022. interview | | The Childbirth Attitudes Questionnaire (CAQ),  The Perceived Social Support Scale (PSSS), Connor-Davidson Resilience Scale (CD-RISC), General Self-efficacy Scale (GSES) | -Resilience could explain 41.6% and fear of childbirth 33.1% of psychological distress | -relieving psychological distress level | | high resilience associated with low fear of childbirth | Pregnant women with high resilience-low fear of childbirth had significantly lower levels of psychological distress than those with low resilience-high fear of childbirth. The indirect effect of fear of childbirth on psychological distress through resilience was significantly.  The interactions between fear of childbirth and adverse childhood experiences and between resilience and adverse childhood experiences were significant |
|  | | Oliwia G et al.,2023  COVID | Polish, 17 February to 13 October 2021, longitudinal study, using social media, 122 perinatal women. | | Edinburgh Postnatal Depression Scale (EPDS), Beck Depression Inventory (BDI - 2), Resilience Measure Questionnaire (KOP26) | 26.2% had depressive symptom | Psychoeducation | | Low resilience was significantly associated with depressive symptoms and anxiety related to childbirth. | Findings highlight the importance of considering resilience as an important factor in understanding and managing perinatal depression and may have implications for the development of targeted interventions. |
|  | | Yumei Shi et al.,2022 | China, Cross sectional study, 579 pregnant women, December 2021 to April 2022, self-filled questionnaires | | Chinese Pregnancy-related Anxiety scale, Connor-Davidson Resilience Scale, Multidimensional Scale of Perceived Social Support | Pregnancy related anxiety 41.4% | Family  function and perceived social support | | Resilience and perceived pregnancy related anxiety have inverse relationship | - perceived social support and family function factors for resilience. |
| Mixed | | | | | | | | | | |
|  | Jacqueline A. et al 2021 | | Australia, perinatal women, COVID-19 Quantitative: 174 ( 31 pregnant and 143 were postpartum- up to one-year post-birth)  Qualitative: 14 interviews using Purposive sampling Qualitative: online using Semi-structured interviews  Quantitative: online developed non validated tool | Mental Health Continuum—Short Form (MHC-SF), mindfulness Attention Awareness Scale (MAAS), Self-Compassion Short Form Scale (SCS), Perceived Stress Scale (PSS). | | perceived stress & wellbeing | mindfulness  and self-compassion | The relationship between mental health and resilience revealed positive association. resilience traits and positive mindsets may be protective against psychological distress for the mother and her child | | Meditation-based or similar training for expectant women might help support resilience them during times of crisis, such as a pandemic. |
|  | Emma C et al 2020 | | sample of Aboriginal  women’s, Australia  pregnant (more than 6 weeks gestation) & had a child aged between 7 days and 12 months, Non-COVID-19 (2013–2014), qualitative - Sample (91) using retrospective survey. Quantitative- not explained | Kimberley Mum’s Mood Scale (KMMS) | | Depression and/or anxiety (25%) | Family-based support (mothers followed by sisters), self-care | Protective –family, healthy lifestyle ,emotional self-regulation, good childhood experience  Risky - loss and grief (managed by family support), IPV. | | family as stress, lack of  emotional regulation/self esteem and intimate partner violence were individual significantly associated with higher KMMS risk and having clinical depression and/or anxiety. Disclosure of IPV, experienced childhood adversities, was significantly associated with a diagnosis of a depression and anxiety. |
|  | Caroline Sh et al.,2022 | | USA, women during 2–6 months, postpartum,  Quantitative=medical record review and an electronic survey & Semi-structured individual interviews for qualitative | Quantitative= Brief Assessment of Recovery Capital (BARC-10), qualitative= interview guide based on the (Substance Abuse and Mental Health Services Administration (SAMHSA)’s recovery framework served). | | Opioid Use Disorder | Recovery | Consistent with SAMHSA’s framework, our participants’  Substance use management improved in recovery and enabled recovery progress. | | Recovery goals included = no use of drugs or alcohol (62.5%), being a better partner/spouse (87.5%), and improving finances (87.5%).  Qualitative = recovery  as transformative, building resilience, and transforming one’s health, relationships, and environment through recovery. |
|  | Patricia A et al 2021 | | USA, n =524, cross-sectional observational study survey, pregnant and postpartum (up to 6 months post-delivery) women. During COVID-19 pandemic from April–June, 2020 , Online data collection method, | Brief Symptom Inventory-18 (BSI), post-traumatic stress disorder (PTSD) checklist for DSM-5 (PCL-5), Connor-Davidson Resilience Scale (CD-RISC 2), Coronavirus Perinatal Experiences Impact Survey (COPE-IS). | | depression,  Anxiety and post-traumatic stress disorder. | Social support and self-care | Women with family and job concerns and low resilience/ adaptability scores seem to be at high risk of psychological sequelae. Use of social media is thought to improve social connectedness, our results indicate that increased media consumption is related to increased anxiety symptoms. | | Determinants of outcome variable: Quantitative: job insecurity, family concerns,  eating comfort foods, resilience/adaptability score, sleep, and use of social and news media. Qualitative themes pervasive uncertainty and anxiety; grief about losses; gratitude for shifting priorities; and use of self-care methods including changing media use. Commonly utilized self-care practices: changing their relationship with technology to maintain support with friends, family members, and healthcare providers) and engaging in self-care activities (e.g., exercise, time outdoors, eating well, prayer)). |
|  | Charlotte V et al.,2020 | | USA, 31 pregnant and postpartum women, During (COVID-19) pandemic, online survey, | Patient Health Questionnaire–2 (PHQ-2), Generalized Anxiety Disorder–7 (GAD-7),  Brief Resilience Scale (BRS), Warwick-Edinburgh Mental Wellbeing Scale (WEMWBS),  Loneliness Scale, | | 12% of the high depressive symptom and 60% reported moderate or severe anxiety. 40%of the sample reported being lonely | Self-care  Being outdoors, gratitude, adhering to routines | Various resilience mechanisms found to have positive effect on the mental health condition. | | Qualitative data suggested that social support, and specifically partner and emotional support, gratitude and optimism and the management or shifting of expectations were significant protective factors for pregnant and postpartum women, particularly during exposure to significant environmental stressors. Quantitative: use of virtual communication platforms, engaging in self-care behaviors, partner emotional support, being outdoors, gratitude, and adhering to structures and routines. |

**Table 1.** Characteristics of included studies
